# 3D Electrospun Nanofiber-Based Scaffolds: From Preparations and Properties to Tissue Regeneration Applications

**DOI:** 10.1155/2021/8790143

**Published:** 2021-06-17

**Authors:** Shanshan Han, Kexin Nie, Jingchao Li, Qingqing Sun, Xiaofeng Wang, Xiaomeng Li, Qian Li

**Affiliations:** ^1^School of Mechanics and Safety Engineering, Zhengzhou University, Zhengzhou 450001, China; ^2^National Center for International Joint Research of Micro-nano Moulding Technology, Zhengzhou University, Zhengzhou 450001, China; ^3^School of Chemical and Biomedical Engineering, Nanyang Technological University, Singapore 637457, Singapore; ^4^Center for Functional Sensor and Actuator, National Institute for Materials Science, 1-1 Namiki, Tsukuba, Ibaraki 305-0044, Japan

## Abstract

Electrospun nanofibers have been frequently used for tissue engineering due to their morphological similarities with the extracellular matrix (ECM) and tunable chemical and physical properties for regulating cell behaviors and functions. However, most of the existing electrospun nanofibers have a closely packed two-dimensional (2D) membrane with the intrinsic shortcomings of limited cellular infiltration, restricted nutrition diffusion, and unsatisfied thickness. Three-dimensional (3D) electrospun nanofiber-based scaffolds can provide stem cells with 3D microenvironments and biomimetic fibrous structures. Thus, they have been demonstrated to be good candidates for *in vivo* repair of different tissues. This review summarizes the recent developments in 3D electrospun nanofiber-based scaffolds (ENF-S) for tissue engineering. Three types of 3D ENF-S fabricated using different approaches classified into electrospun nanofiber 3D scaffolds, electrospun nanofiber/hydrogel composite 3D scaffolds, and electrospun nanofiber/porous matrix composite 3D scaffolds are discussed. New functions for these 3D ENF-S and properties, such as facilitated cell infiltration, 3D fibrous architecture, enhanced mechanical properties, and tunable degradability, meeting the requirements of tissue engineering scaffolds were discovered. The applications of 3D ENF-S in cartilage, bone, tendon, ligament, skeletal muscle, nerve, and cardiac tissue regeneration are then presented with a discussion of current challenges and future directions. Finally, we give summaries and future perspectives of 3D ENF-S in tissue engineering and clinical transformation.

## 1. Introduction

Tissue engineering that combines cells, biomaterials, and biochemical and biophysical factors to improve or replace biological tissues provides an ideal treatment option for tissue damages [1, 2]. It involves isolation and expansion of target cells *in vitro* and their seeding and growth in implanted biomaterials to allow the formation of new tissues with defined shapes and functions [3]. By utilizing these autogenous cells, tissue engineering has the advantages of autografts, which overcomes the limited self-repairing capacity of many tissues [4]. To date, this therapeutic approach has demonstrated success in the repair of skin, cartilage, bone, bladder, and blood vessels, among others [5–9].

Biomaterials play essential roles in tissue engineering as they can provide designable biophysical and biochemical milieus to support cell attachment, proliferation, differentiation, and neo tissue genesis [10–12]. With the increasing understanding of the interactions between cells and the surrounding microenvironment, more and more attention is focused on developing biomaterials that mimic ECM's native components, properties, and structures [13–15]. An optimal scaffold for tissue regeneration should mimic the mechanical and functional properties of ECM of those tissues to be regenerated. To date, a significant number of biomaterials, including hydrogels, porous scaffolds, and fibers, have been extensively developed and explored in tissue engineering.

Among them, fibrous scaffolds have recently attracted a lot of attention since native ECM consists of abundant protein fibers with different structures and arrangements [1, 16]. Electrospun nanofibers with controllable diameters, alignments and components, and large surface areas are often used to meet the requirements of tissue engineering [17–21]. However, these traditional 2D electrospun membranes have an intrinsic limitation of relatively poor cellular infiltration due to their limited thickness and relatively high packing density [22, 23]. Therefore, these scaffolds, in practice, act as 2D surfaces rather than 3D microenvironments. Although porous scaffolds and hydrogels can provide a suitable 3D microenvironment for implanted cells and have exhibited extensive applications in regenerative medicine [24–26], they often lack fibrous structures and the anisotropy character of native tissue ECM [27, 28]. Thus, researchers are seeking to develop 3D electrospun nanofiber scaffolds to recapitulate ECM's architecture and morphology better [29].

**Figure 1:**
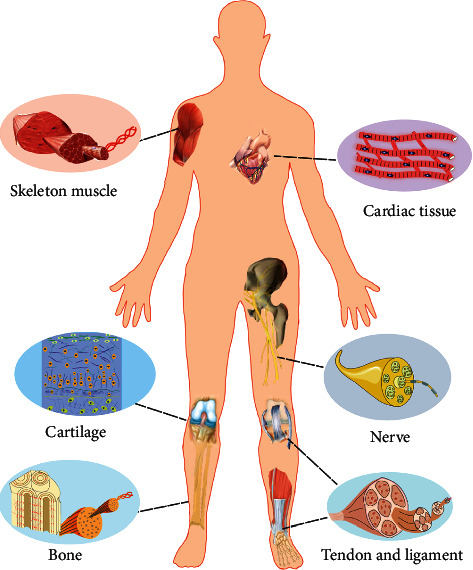
Illustration of typical tissues with fibrous structures whose regeneration can be mediated by 3D ENF-S.

There are some review articles about electrospinning and nanofiber-hydrogel composite scaffolds [30–32]. However, a comprehensive summary of fabrication strategies, required functions, and advanced performances in tissue engineering is missing. This review article summarizes the recent design approaches of 3D ENF-S and their applications in tissue engineering. It starts with a brief introduction to the traditional electrospun nanofibers and their limitations. Subsequently, recent advances in the fabrications of three types of 3D ENF-S are presented. The applications of 3D ENF-S with improved features and functions in various types of tissue engineering are then highlighted (Figure 1). Finally, we give a brief conclusion and discuss the perspectives on current challenges and future directions of 3D ENF-S in tissue engineering.

## 2. Preparation of ENF-S from 2D to 3D

### 2.1. Traditional Electrospinning and Its Limitation

Electrospun nanofibers are often fabricated using electrospinning facilities which consist of three elements: an electrical generator (high voltage supply), a needle with or without solvent pump (jet source), and a metal collector (target) [33]. When voltage is applied on the nozzle and electrostatic repulsion counteracts the surface tension, the droplet will be stretched. At the critical voltage point (the threshold voltage), a “Taylor cone” (disintegration of water drops in an electric field) is formed, and a jet of liquid erupts from the surface. The jet flow created at the “Taylor cone” is stretched to whip into continuous ultrafine fibers in the electric field while the solvent is evaporating. By adjusting experimental parameters such as the applied voltage, the designated distance between the nozzle and collector, and the property of the solution, fibers with uniform diameters can be routinely produced, and an electrospun membrane with packed density is gradually fabricated. The nanofibers' diameters and orientations can be controlled by adjusting the solution viscosity or the type of collectors, which leads to an apparent influence on cell behaviors and functions [34, 35]. However, electrospun membrane produced by traditional strategy possesses a very thin thickness (<1 mm) [36] and high dense packing attributed to the nature of electrospinning, which hampers cell infiltration, and limits extensive applications in tissue regeneration.

### 2.2. Preparation of 3D Electrospun Nanofiber-Based Scaffolds (ENF-S)

**Figure 2:**
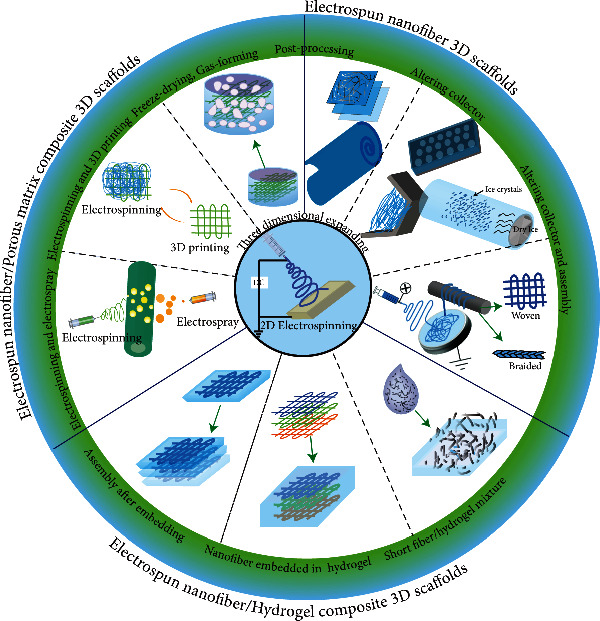
The summary of representative design approaches of 3D ENF-S.

3D scaffolds are required for most tissue regeneration because their biomimetic 3D environment will promote cell differentiation, neo tissue development, and higher genetic material expression, such as ECM secretion and cell metabolism. Therefore, 3D ENF-S with thick thickness and the capacity for cell infiltration is highly needed for tissue engineering. In this section, 3D ENF-S are classified into (i) electrospun nanofiber 3D scaffolds, (ii) electrospun nanofiber/hydrogel composite 3D scaffolds, and (iii) electrospun nanofiber/porous matrix composite 3D scaffolds according to the different fabrication approaches (Figure 2).

#### 2.2.1. Electrospun Nanofiber 3D Scaffolds

Electrospun nanofiber 3D scaffolds with high porosity and thickness can be fabricated by direct electrospinning through postprocessing techniques, tuning fiber collection techniques, or combining the two. Electrospun membranes can be changed into scaffolds with desirable 3D structures, tailored sizes, and mechanical properties by facile and direct postprocessing techniques such as stacking, rolling, and braiding the electrospun membrane [37]. Besides, gas foaming is also successfully used to expand 2D electrospun nanofiber sheets to 3D scaffolds. For example, electrospun nanofiber mats were successfully grown into a 3D scaffold after treatment with NaBH_4_ aqueous solutions [38]. The distributions of gap widths and layer thicknesses are directly dependent on nanofiber mats processing time within the gas bubble forming solution. Similarly, a porous poly(*ε*-caprolactone) (PCL) electrospun structure was fabricated by adding a chemical blowing agent, azodicarbonamide, which generated micro-sized pores through decomposition after deposing at 100°C for 2-3 seconds [39]. The montmorillonite-reinforced poly(L-lactide) (PLLA) electrospun nanofibers with a 3D and dual-porosity structure were fabricated through a cold compression molding process and salt leaching/gas-forming method [40]. Other postprocessing methods, such as hole forming by punching or laser ablation, can enhance electrospun mats' porosity and promote tissue regeneration performance by improving cell infiltration and migration [41, 42].

Electrospun nanofiber 3D scaffolds with significantly increased thickness can also be prepared by tuning fiber collection techniques, such as regulating the electrospinning collector or the environment during electrospinning. According to a study conducted by Subramanian et al., a 3D tubular scaffold was fabricated by adding an insulating gap as an auxiliary electrode, which resulted in a longitudinal deposition of fibers [43]. Similarly, spherical dish and double-bevel collectors were also used to fabricate 3D fibrous scaffolds [44, 45]. Patterned collectors could increase the thickness and pore size of electrospun nanofiber scaffolds to improve cell infiltration [46–50]. Besides the geometry of the collector, Olvera et al. found that electrospun nanofiber 3D scaffolds could be fabricated readily by varying the rotational speed of the mandrel collector during electrospinning [51]. Moreover, cryogenic electrospinning was also used to fabricate electrospun nanofiber 3D scaffold with high porosity and thickness utilizing ice crystal as templates [52–54]. For instance, a poly(D, L-lactide) scaffold with large pores ranging from 10 to 500 *μ*m was fabricated by the cryogenic electrospinning technique [55]. Besides, centrifugal electrospinning, using centrifugal forces to produce fibers from melts or solutions, has also been reported to produce 3D fibrous scaffolds. This technology is high throughput, and the scaffolds prepared by this technology have open porous structures facilitating deep cell penetration [56, 57].

Tuning collectors and postprocessing techniques are often combined to increase further the thickness and porosity of electrospun nanofiber scaffolds. For example, 3D porous electrospinning PCL with nanohydroxyapatite (nHA) scaffolds were prepared using the stainless-steel porous mesh collector and layer-by-layer (LbL) assembly technique [58]. Another popular strategy is the electrospinning-based yarn assembly technique, forming yarns by regulating the collection system and further postprocessing by 3D assembling [59–61]. For example, the yarns obtained through a liquid support system can produce 3D scaffolds with different shapes [62]. Similarly, yarns can be collected through an “electro-wet spinning,” in which a water vortex is formed to twist the collected fibers into yarns in a dynamic liquid collection system [63, 64]. A combination of this hydroelectrospinning and LbL assembly can produce 3D scaffolds [65]. Further, the yarn-based 3D scaffolds could be prepared by postprocessing treatment [66]. Moreover, the yarns can be used to weave 3D anisotropy scaffolds, which exhibited excellent anisotropy mechanical properties for specific tissue regeneration [67].

#### 2.2.2. Electrospun Nanofiber/Hydrogel Composite 3D Scaffolds

Hydrogels have been utilized for various biomedical field applications, including drug loading, cell delivery, and tissue engineering, because of their 3D spatial architecture and elastic properties [68–70]. However, hydrogels have poor mechanical strength and lack native ECM's fibrous structure [31]. The amalgamation of electrospun fibers and hydrogels can simulate the structures and properties of ECM [71]. Nanofiber components can increase cell viability, adhesion, and differentiation because the nanofibers can resist the contractile forces, providing adhesion sites and maintaining the oriented morphology [72]. The mechanical properties and cell in infiltration ability of such composite constructs are significantly enhanced compared to the individual counterparts [73]. The design approaches to integrate hydrogels with electrospun nanofibers will be summarized in this part. Simultaneously, their roles and synergy functions in various tissue regeneration will also be emphasized.

Electrospun nanofiber/hydrogel composite 3D scaffolds can be fabricated by embedding the postassembled electrospun nanofibers in hydrogel precursor solution or assembled after combining electrospun nanofibrous membranes and hydrogels. For example, electrospun PLA nanofiber sheets were coated with poly(lactide-co-ethylene oxide fumarate) (PLEOF) hydrogels. They were assembled into composite scaffolds through the LbL assembly and crosslinking. In this approach, the precursor solution acted as a “glue” to hold the fiber layers together [74]. The formed laminated or rolled nanofiber/hydrogel composite scaffolds possess significantly higher mechanical strength than the pure hydrogels [74, 75]. Besides, the embedded nanofibers were reported to improve hydrogels' mechanical and biological properties, such as the ability to act against contraction or degradation during tissue regeneration and provide the attachment signal and directional cues to cells [72]. Also, the interaction between nanofibers and hydrogels would affect the performance of final products [76].

Short nanofibers via cutting or degrading the electrospun nanofibers can be suspended in hydrogel precursor solutions before gelation [77, 78]. This integration process retains the injectability of the constructs. For instance, gelatin/PCL electrospun short fibers were mixed with gelatin solution and then crosslinked with glutaraldehyde to prepare composite scaffolds. These composite scaffolds showed more robust mechanical properties than pure hydrogels due to the strong interfacial bonding between the nanofibers and the hydrogels [79, 80]. Short electrospun nanofiber/hydrogel mixture could also be processed into bioinks suitable for 3D bioprinting to fabricate constructs with complex structure and improved mechanical properties [81].

#### 2.2.3. Electrospun Nanofiber/Porous Matrix Composite 3D Scaffolds

Scaffolds with interconnected porous structures are attractive for tissue engineering because the porous structures provide spaces for cell migration, proliferation, nutrition/waste transportation, and neo tissue regeneration [12, 25]. It was reported that fibrous structures in porous scaffolds could further accelerate cell adhesion and migration due to their similarities with native ECM [82]. Therefore, many recent studies have introduced nanofibers into porous scaffolds. The methods, such as freeze-drying, electrospray, and 3D printing, for porous scaffold fabrication, can be integrated with electrospinning to make nanofiber-based 3D scaffolds. Therefore, combining electrospinning with other 3D porous structure forming technologies is also an effective approach to fabricate 3D ENF-S.

3D ENF-S with porous structures can be easily formed by freeze-drying. For example, regenerated cellulose (RC) and PLA nanofibrous composite scaffolds were fabricated by freeze-drying and followed by crosslinking with citric acid. These RC/PLA composite scaffolds with dual pore structures exhibited superabsorbent, good stability, and mechanical property [83]. Similarly, bilayer collagen-nanofiber composite porous scaffolds were prepared by compression and freeze-drying. There is no apparent gap between the porous and nanofiber layer, showing a good integration [84]. Poly(ethylene oxide) (PEO) microparticles and PLA nanofibers produced by electrospray and electrospinning, respectively, are deposited on the collector simultaneously to form the nanofiber/microparticle composite scaffolds. The thickness can significantly increase after introducing microparticles, which can release electric charge and build the construct during electrospinning [85, 86].

3D printing is a frequently used method to fabricate porous scaffolds, especially for scaffolds with complex structures. Combined with 3D printing, the dimension of the structure and the scale of electrospun nanofibers pore will increase from nanoscale to the macroscale resembling the ECM's topographic characteristic [87]. Yu et al. fabricated a composite 3D scaffold by printing PCL grid scaffold with the macroscale pores as the fundus and infusing PCL/gelatin short nanofibers into the printed scaffolds [88]. 3D-printed PCL grid was reported to have a smooth surface, while electrospinning PCL nanofibers connected by simply gluing improved the chondrocyte viability, adhesion, and infiltration [89]. The short nanofibers were also used for porous and fibrous scaffold fabrication. For example, a semifluid mixture of HA/PEO solution and gelatin/poly(lactide-co-glycolide) (PLGA) electrospun short nanofibers working as a novel ink was extruded to fabricate 3D-printed fiber-based scaffolds. By tuning parameters (the inner diameters of the nozzle, the moving speed of the plotting head, and the dosing speed) and adjusting the distance and diameters of the strands, the composite scaffolds with different shapes and pore sizes can be achieved [90].

Moreover, direct polymer melt deposition was also combined with electrospinning to fabricate 3D scaffolds with fibrous structures. Park et al. deposited the electrospun polycaprolactone/collagen nanofibers between the polycaprolactone microfiber layers prepared by direct polymer melt deposition to form 3D ECM-like tissue engineering scaffolds. This nano/microfiber composite 3D scaffold provided the optimal environment for chondrocyte adhesion and proliferation due to the improvement of biocompatibility and inner surface's enlargement [91]. Other approaches, such as selected laser sintering [92] and stereolithography [93], were also used to fabricate tissue engineering scaffolds with 3D porous and fibrous structures, which can accelerate cell infiltration and tissue regeneration [94].

## 3. Advanced Functional Properties of 3D ENF-S

3D ENF-S with cell-permeable structure and controllable thickness is more practical than 2D electrospun membrane in most tissue regeneration. As a critical factor in tissue engineering, the tissue engineering scaffold should substitute for the native ECM while mimicking its structure and mechanical properties. Compared with other kinds of scaffolds, such as porous or hydrogel scaffolds, these 3D ENF-S have also exhibited many other advanced functional properties, such as 3D fibrous architecture and tunable mechanical properties, making them a focus of recent studies.

### 3.1. 3D Fibrous Architecture

It is widely accepted that the creation of biomimetic niches closely resembling the native biological environment is critical to guide cell growth and differentiation and promote target tissue regeneration. Although there are already many studies involving bioinspired ECM design, related studies are still developing rapidly [95]. 3D ENF-S has drawn extensive attention due to its nanofibrous structures, which can resemble the various tissues of native ECM's fibrillar features [96]. This fibrous structure has been demonstrated to play a vital role in regulating how cells interact with the native ECM, influencing cell adhesion, spreading, proliferation, and differentiation. Therefore, 3D ENF-S with these biomimetic fibrous structures have been developed for various kinds of tissue regeneration.

### 3.2. Mechanical Properties

Scaffolds' mechanical properties are essential for their successful performance in tissue regeneration. Considering the *in vivo* implantation and clinical application, mechanical strength and suture retention strength are vital for tissue engineering scaffolds. Among the different types of scaffolds, 3D ENF-S with excellent and adjustable mechanical properties have great potential for clinical transplantation.

#### 3.2.1. Mechanical Strength

Scaffolds must be sufficiently strong to match the mechanical properties of the native tissue at the implantation site to support cell adhesion, cell spreading, and ECM synthesis. Additionally, when applied *in vivo*, the scaffold must withstand external forces acting upon it to maintain its integrity and support its function without collapsing. Recently, many studies reported that 3D electrospun nanofiber-based scaffolds with outstanding mechanical properties have been successfully applied in tissue engineering, especially in force loading tissues, such as cartilage, tendon, and ligament [97, 98]. Biomimicing the collagen nanofibers in native tissue, aligned electrospun nanofibers have exhibited extraordinary mechanical strength, enhancing tissue engineering scaffolds' mechanical properties [60]. In composite scaffolds, electrospun nanofibers can improve mechanical strength through strain transfer between the matrix and the nanofiber reinforcement.

#### 3.2.2. Suture Retention Strength

Suture is a common method to fix implants to surrounding tissues [99]. The limited suture ability of scaffolds may hinder the in situ implantation and then impede the *in vivo* experiments and clinical transplantation, especially for the tissues or organs that require high and dynamic loads, such as vascular, tendon, and ligament [100]. However, few studies on engineered scaffolds measure suture retention strength, despite its established importance for clinical implementation. 3D ENF-S presents suture ability and enough suture retention strength for *in vivo* implantation. For example, Vaquette et al. braided electrospun PCL mesh with cell seeding to develop a tissue-engineered ligament construct that can be sutured with the surrounding tissue [101].

### 3.3. Structural and Mechanical Anisotropy

Anisotropy, which refers to the directional dependence of physical properties, is one fundamental property of some tissues, such as cartilage, muscle, tendon, and ligament [27, 102, 103]. Anisotropy gives ECM the ability to maximize its function along the direction of use. For instance, these unique structures can impose effective force transmission and contractility for the regeneration of functional muscle fibers [104–106]. Anisotropic scaffolds with structural and mechanical anisotropy are highly needed to mimic the target tissue properties and provide a specific microenvironment for cell differentiation. Many 3D ENF-S with required anisotropy properties have been prepared by controlling electrospun nanofibers' alignment and arrangement [107, 108].

### 3.4. Degradability

One of the requirements of tissue engineering scaffolds is biodegradation. Degradation itself is a regulatory factor that controls cell behavior and differentiation [2]. Biomaterials can undergo degradation through ester hydrolysis, enzymatic hydrolysis, or photolytic cleavage of the polymer chains *in vitro* and *in vivo*. Based on these mechanisms, scaffolds with good biodegradability and ideal degradation rate can be designed as temporary supports and gradually degraded and replaced by regenerated tissue. Generally, natural polymers, such as gelatin and collagen, have excellent biocompatibilities but a rapid degradation rate. Most synthetic polymers lead to mechanically stable electrospun nanofibers. Electrospun nanofiber composite scaffolds with tailored degradation rates can be designed for tissue engineering applications [52, 74].

Based on the functional properties discussed above, it is found that 3D ENF-S have shown substantial advantages in architecture and mechanical properties compared with other types of scaffolds, thus shortening the distance for clinical applications. However, most studies have performed mechanical tests before implantation. In a dynamic microenvironment *in vivo*, scaffolds will exhibit significantly different performance, such as rapid degradation. Therefore, changes in the mechanical properties of 3D ENF-S under *in vivo* environment are particularly worthy of attention, especially for heavy-duty tissues, such as muscle, tendon, and ligament.

## 4. Tissue Regeneration Application

### 4.1. Cartilage and Bone

**Figure 3:**
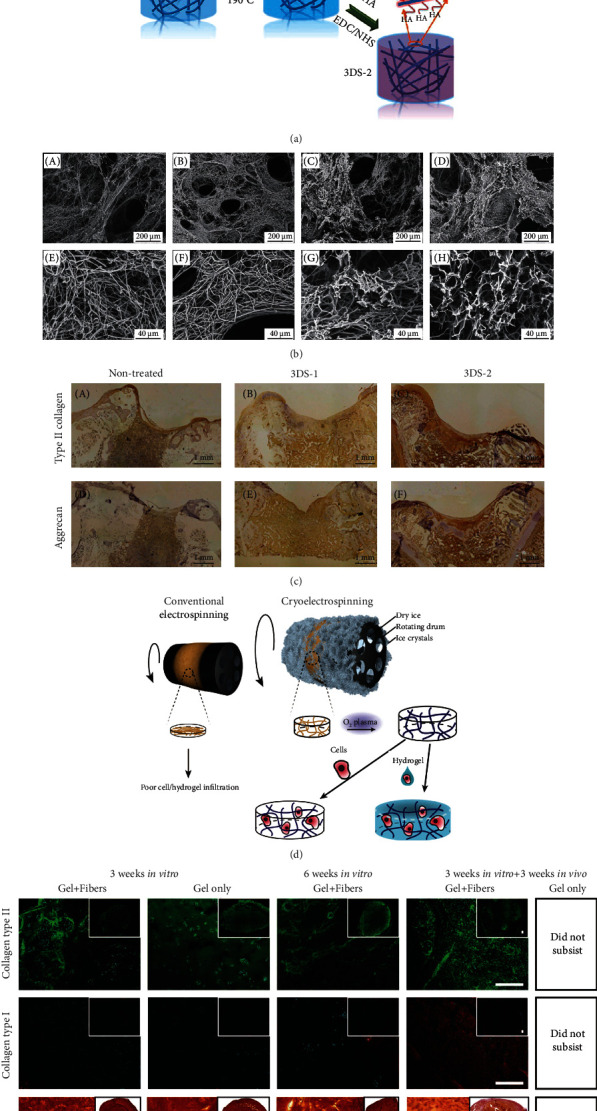
3D electrospun nanofiber-based scaffolds for cartilage tissue regeneration. (a) The schematic illustration for fabrication and crosslinking of electrospun nanofiber porous 3D scaffold (3DS-1) and crosslinked with hyaluronic acid scaffold (3DS-2). (b) The porous and fibrous structure of uncrosslinked (A, E), heat-treated (B, F), crosslinked 3DS-1 group (C, G), and 3DS-2 group (D, H). (c) Collagen type II and aggrecan immunohistochemical staining results of nontreated, 3DS-1, and 3DS-2 scaffolds after *in vivo* implantation for 12 weeks. Reproduced with permission from [115]. Copyright © 2021, American Chemical Society. (d) Conventional electrospinning formed dense nanofiber membrane. Cryoelectrospinning (on a mandrel collector at -78°C) induced 3D porous PCL scaffold due to the ice crystal formation. (e) Hydrogel/nanofiber composite constructs exhibited good chondrogenic ECM deposition and higher stability than pure hydrogel scaffold *in vitro* cell culture and *in vivo* implantation. Reproduced with permission from [52]. Copyright © 2021, WILEY-VCH Verlag GmbH & Co. KGaA, Weinheim.

Osteoarthritis commonly known as the cartilage defect is a disease induced by traumatic injury or degenerative joint diseases. With an aging population and the growing problem of obesity, the number of osteoarthritis cases is estimated to multiply in the future [109]. The popular treatments for articular cartilage repair include microfracture, autologous chondrocyte transplantation, and osteochondral allograft transplantation [110]. Although these techniques have successfully relieved pain and improved joint function, their disadvantages restricted their long-term clinical application [111]. Cartilage tissue engineering as a promising strategy has attracted considerable efforts in the past several decades [112]. Recently, electrospun nanofibers have been used for cartilage tissue engineering due to their nanofibrous network structure [113, 114]. Chen et al. prepared gelatin/PLA nanofiber-based 3D porous scaffold by using nanofiber membrane suspended solution through freeze-drying and heating processes to fabricate a 3D nanofibrous scaffold and overcome the limitations of commonly produced electrospun nanofiber 2D membrane. This type of scaffold could be crosslinked with hyaluronic acid using 1-ethyl-3-(3-dimethyl aminopropyl) carbodiimide (EDC)/N-hydroxysuccinimide (NHS) to promote the function for cartilage regeneration further [115]. The composite scaffold promoted cartilage regeneration, indicated by positive collagen type II and aggrecan immunohistochemical staining results after implantation *in vivo* for 12 weeks (Figures 3(a)–3(c)). Electrospun nanofiber/porous matrix scaffolds also exhibited good performance in cartilage tissue regeneration [116, 117]. For instance, the collagen-poly(vinyl alcohol) nanofibers were electrospun on the surface of a freeze-dried porous collagen sponge to fabricate a composite scaffold, which was designed to replicate the superficial and transitional zones of articular cartilage [118].

Cartilage ECM consists of fibrous collagen II and proteoglycan-based ground substance. Therefore, it was believed that fiber/hydrogel composite 3D scaffold could mimic both the structure and function of native cartilage ECM [119]. For example, a cooled mandrel collector at -78°C was used to prepare electrospun PCL nanofibers to mimic the fibrillar component of cartilage. With the ice crystallization process, the deposited PCL fibers exhibited loose fibrous structure and increased thickness. After sublimation, the macropores are created to allow cell infiltration. The O_2_ plasma treatment was used to make the fibers with a hydrophilic surface, ensuring good contact with cell-laden alginate hydrogel. The cryoelectrospun PCL fiber scaffolds with a thicker thickness (1.5 mm) could enhance alginate hydrogels' mechanical property and stability. After three weeks of *in vitro* culture and three weeks *in vivo* implantation, such a hydrogel/fiber composite scaffold still existed and showed many chondrogenic ECM deposition while the pure hydrogel scaffold was degraded (Figures 3(d) and 3(e)) [52].

Bioactive hydrogels' insufficient mechanical property is a severe challenge in cartilage tissue engineering [97]. Nanofiber/hydrogel composite scaffolds with an improved mechanical property and a chondrocyte preferred 3D microenvironment have become a promising candidate [98, 120]. Sharifi et al. fabricated a composite scaffold using fragmented electrospun PLA fibers and alginate-graft-hyaluronate hydrogel. The compressive modulus of such a composite scaffold increased by 81% compared with hydrogel without mixing with nanofibers. The chondrocytes cultured in this composite scaffold showed a round cell shape and produced a cartilage-specific matrix [121]. The mechanical property and the stability of 3D-printed alginate hydrogels were improved after reinforcement with PLA submicrofibers [81].

The interaction between fiber and hydrogel matrix is essential for chondrogenesis in electrospun nanofiber/hydrogel composite scaffolds. For example, silk fiber (SF) enhanced chondrogenesis when embedded in silk hydrogel rather than standard agarose hydrogel. The newly synthesized proteoglycan was found to be around the silk microfibers in SF-silk hydrogel. In contrast, poor stress transfer and ECM deposition occurred in SF-agarose hydrogel without fiber/hydrogel binding [122]. The nanofiber component can also accelerate the proliferation and secretion of chondrogenic ECM by tuning the constructs' structural morphology and chemical composition [123].

**Figure 4:**
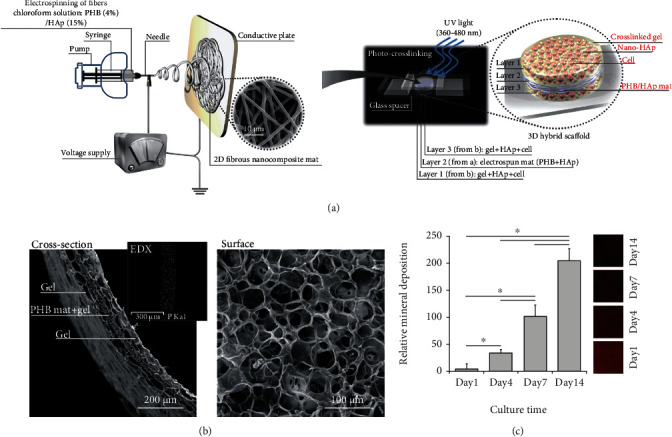
Electrospun nanofiber/hydrogel 3D scaffolds for bone tissue regeneration. (a) Schematic diagram of the experimental steps and the structure of 3D cell-laden hybrid scaffolds. (b) SEM micrographs of surface and cross-section of the freeze-dried hybrid scaffold. (c) Gross appearance of Alizarin Red staining and mineral quantification of hybrid scaffolds after incubation in SBF at 37°C for specific periods (mean ± SD of six replicates). Reproduced with permission from [124]. Copyright © 2016, Elsevier B.V.

Bone tissue engineering also requires a scaffold with extremely high mechanical strength. The combination of electrospun nanofibers is a practical approach to increase mechanical strength and provides an option for using hydrogel as a scaffold for bone tissue engineering. Shojai and coworkers prepared a trilayered scaffold that consisted of polyhydroxybutyrate (PHB), hydroxyapatite (HA), and gelatin methacryloyl (GelMA) hydrogel for bone tissue engineering (Figures 4(a) and 4(b)). In their work, electrospun PHB mats were punched and soaked in GelMA precursor solution with suspended HA nanoparticles for 20 min, which was then sandwiched between GelMA with HA nanoparticle layers. The 3D nanofiber/hydrogel composite scaffold was formed by UV crosslinking. Compared with electrospun fiber or hydrogel alone, this trilayered scaffold exhibited the required mechanical strength and superior microenvironment. Also, the matrix mineralization and alkaline phosphatase (ALP) activity were improved in this hybrid scaffold due to the presence of HA nanoparticles (Figure 4(c)) [124]. Lamination of PLA fiber/PLEOF hydrogel composite scaffold was fabricated by LbL assembly and further crosslinking of hydrogel component. The fiber mesh gave this composite scaffold strong mechanical strength, and the hydrogel components connecting these different layers accelerated the diffusion of oxygen and nutrients.

Furthermore, with hydrogel degradation, there will be more space for cell proliferation and ECM deposition. After combining HA nanocrystal and Arg-Gly-Asp (RGD) peptide in the hydrogel phase, this laminated composite scaffold promoted the osteogenic differentiation, which was confirmed by detecting higher ALP activity and expressing osteogenic markers (osteopontin and osteocalcin) [74]. Similarly, Naghieh et al. produced hierarchical scaffolds stacked with PLA microfiber layers prepared by fused deposition modeling and nanocomposite gelatin-forsterite fibrous layers fabricated through electrospinning. The elastic modulus of this composite scaffold was increased by more than 1.5-fold compared with the PLA scaffolds without the combination of electrospun fibers. After soaking in a simulated body fluid (SBF) solution for 28 days, HA crystals were found on the struts of the composite scaffold, indicating a bioactive candidate for bone tissue regeneration [125].

**Figure 5:**
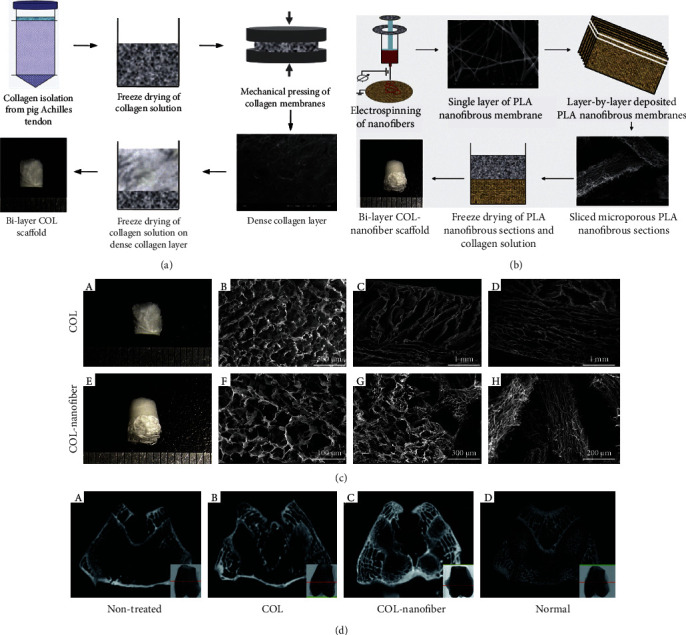
Electrospun PLA nanofiber/freeze-dried collagen bilayer composite scaffold for osteochondral tissue engineering. (a) The fabrication process of freeze-dried collagen bilayer scaffolds and (b) nanofiber/freeze-dried collagen bilayer composite scaffolds, and (c) their microstructures. (d) Architecture evaluation of the repaired tissues after 12 weeks of implantation by *μ*-CT images ((A) nontreated group, (B) freeze-dried collagen bilayer scaffold group, (C) nanofiber/freeze-dried collagen bilayer composite scaffold group, and (D) normal joints). There were abundant subchondral bones formed in the nanofiber-collagen porous scaffold group. Adapted with permission from [84]. Copyright © 2013, Acta Materialia Inc. Published by Elsevier Ltd.

3D ENF-S are also promising for osteochondral tissue regeneration. In the natural osteochondral region, the ingredient and structure vary from bone to cartilage, in which the complex environment cannot be satisfied by a homogeneous scaffold. Previously, collagen porous scaffolds and electrospun nanofibers have been proven to promote cartilage and bone regeneration, respectively [11, 25]. Therefore, a bilayer collagen/PLLA nanofiber composite scaffold was developed for osteochondral tissue repair (Figures 5(a) and 5(b)). The top layer was a freeze-dried collagen porous scaffold, and the bottom layer consisted of nanofiber strips with 100-300 *μ*m pores (Figure 5(c)) [84]. Mesenchymal stem cells (MSCs) cultured on this collagen/nanofiber composite scaffold showed stronger osteogenic differentiation, which was evidenced by the higher expression of osteocalcin (OCN) and runt-related transcription factor 2 (runx2) osteogenic genes. In the rabbit osteochondral defect model, rapid subchondral bone emergence and better cartilage formation were observed in collagen/nanofiber composite scaffold. Further, the subchondral bone with a bridge-like structure was also observed using *μ*-CT, which was expected to contribute to the top cartilage regeneration (Figure 5(d)) [84].

### 4.2. Tendon and Ligament

Tendons and ligaments are tough connective tissues that connect bone to muscle and bone to bone, respectively. They have strong tensile strength due to their dense fibrous microstructure. Therefore, fiber-based composite scaffolds have drawn much attention for tendon and ligament tissue regeneration [126]. For example, aligned electrospun nano/microfiber scaffolds were developed by mimicking the aligned collagen fibrils of native tendon and ligament [127, 128]. Braided or stacked electrospun PLLA and PCL fiber were prepared to meet the clinical requirements of tensile and suture strength [37]. It was indicated that braided scaffolds with substantial mechanical properties could promote tenogenic markers' expression. Stacked scaffolds have better cell infiltration than braided ones, resulting in a higher total cell number and ECM content. Similarly, Vaquette et al. braided electrospun PCL mesh with cell-laden to develop a tissue-engineered ligament construct [101]. This PCL mesh/cell sheet composite construct had a stress/displacement J-curve, similar to that of the native ligament. *In vivo* experiments showed that ECM was distributed homogenously within the scaffolds after incorporating the cell sheet.

**Figure 6:**
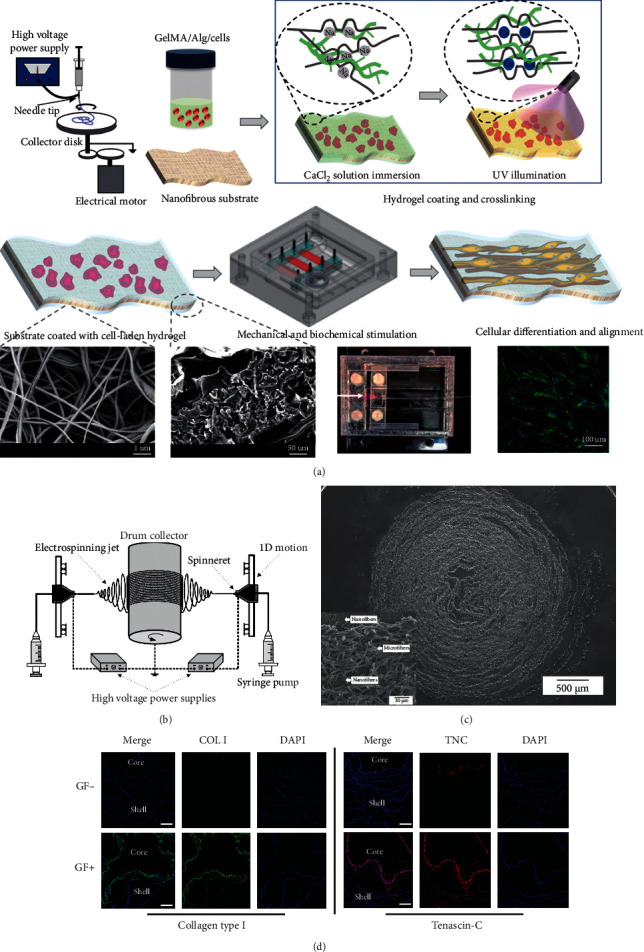
3D electrospun nanofiber-based scaffolds for tendon and ligament regeneration. (a) Schematic illustration of fabrication and stimulation of composite scaffold consisted of electrospun nanofibers and cell-laden hydrogel coating crosslinked by Ca^2+^ and UV light. Reproduced with permission from [129]. Copyright © 2019, American Chemical Society. (b) Electrospinning equipment for fabrication of silk fibroin-PCL nano/microfiber mat. (c) SEM images of cell-laden fiber/hydrogel 3D composite construct (cross-section). (d) Immunofluorescent staining of collagen type I and tenascin-C in the hybrid constructs. Scale bar = 200 *μ*m. Copyright [130].

Electrospun nanofibers and hydrogel composite scaffolds were also selected to achieve the cell-laden and excellent mechanical properties. PCL-polyamide electrospun membranes possessed excellent mechanical properties for tendon replacement but lacked biocompatibility and 3D structure. Therefore, GelMA/alginate thin hydrogel layers were coated on the electrospun nanofibers, providing an ECM-like microenvironment for encapsulated MSCs. Under mechanical stimulation generated by a custom-built bioreactor, the cell viability, proliferation, alignment, and tenogenic differentiation of MSCs were promoted (Figure 6(a)) [129]. Kim et al. also presented a fiber/hydrogel composite scaffold for tendon tissue engineering. The aligned fibers produced by hybrid electrospinning of PCL and silk fibroin provided topological cues for cell alignment and differentiation. Alginate hydrogel with MSCs laden was injected into the fibrous scaffolds after rolling the fibrous layers around a needle to improve cell infiltration and provide a 3D microenvironment. With basic fibroblast growth factor (bFGF) supplements, MSCs exhibited a ligament phenotype, which was approved by the secretion and deposition of related ECM proteins in the composite scaffold (Figures 6(b)–6(d)) [130].

Tendons consist of aligned collagen fibers and surrounding glycosaminoglycan sheath, mainly dermatan sulfate and chondroitin sulfate. Therefore, nanofiber/hydrogel composite scaffolds have also been designed for tendon regeneration. For example, the aligned PLLA nanofibers were manufactured by electrospinning. A chitosan/collagen hydrogel was layered on the nanofiber, rolled into tubes, and then coated with alginate hydrogel to prepare a composite construct. As expected, the mechanical strength of this composite scaffold was significantly enhanced, which was sufficient for the flexor tendon. The aligned nanofibers could guide cell attachment and growth. The hydrogel phase promoted cell infiltration, and the alginate component prevented peritendinous adhesion. The excellent mechanical property and biocompatibility proved that electrospun nanofiber-based composite scaffolds are good candidates for tendon regeneration [75]. The bFGF growth factor and dynamic stimulation were further applied to the braided PCL/collagen fibrous scaffold for tendon regeneration. This composite scaffold with mechanical and biochemical stimulation showed enhanced proliferation, tenogenic marker expression, and aligned collagen morphology after 12 weeks of *in vivo* implantation, further proving the promising application of electrospun nanofiber-based 3D composite scaffold in tendon tissue reconstruction [131].

### 4.3. Skeletal Muscle

Skeletal muscles, comprising between 40 and 45% of an adult human body mass, are mainly responsible for generating force and controlling body locomotion [103]. However, muscle tissues are easily injured. Unfortunately, after severe injuries, the endogenic regeneration of itself is helpless [132]. According to reports, skeletal muscle tissue engineering, designing constructs to meet tissue regeneration's functional and aesthetic requirements in muscle defects, has good prospects [104]. Cellular alignment and elongated myotube are crucial in muscle tissue engineering because skeletal muscle is composed of aligned myofibers and connective tissues [103]. Moreover, scaffolds used to support skeletal muscle regeneration should accommodate and promote the formation of densely packed, highly aligned myofibers, which exert effective force transmission and contractility for the revival of functional muscle fibers [105].

**Figure 7:**
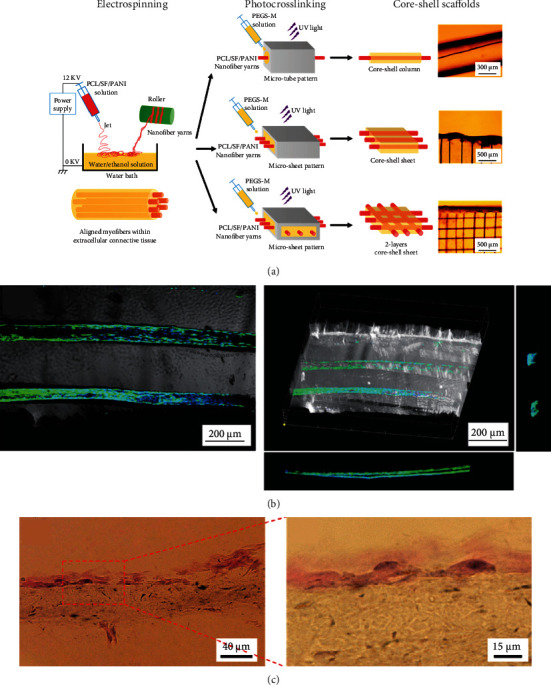
Nanofiber yarn/hydrogel composite 3D scaffolds for myoblast alignment and differentiation. (a) Preparation illustration of yarn/hydrogel composite 3D scaffolds with native skeletal muscle mimicking core-shell structure (aligned nanofiber yarns by electrospinning and PEG-based hydrogel shell via photocrosslinking). (b) Highly organized C2C12 myotubes within the 3D hydrogel. (c) H&E staining images of the construct with C2C12 cell encapsulation after cultivation for seven days. Reproduced with permission from [133]. Copyright © 2015, American Chemical Society.

Recent studies have shown that anisotropic materials may be preferred for developing muscle tissue engineering constructs as their morphology and function are more similar to native tissues [106, 134]. 3D scaffolds with anisotropic properties are needed to provide a 3D microenvironment for implanted cells [134]. Combining hydrogels with electrospun nanofibers offers an excellent solution to this problem. For example, fibers with fibrous 3D bundle structures were fabricated with an electrohydrodynamic jet technique followed by wet electrospinning. After a uniaxial stretch, the fibers become aligned direction from a random structure. Cell encapsulated collagen/PEO bioink was printed on this 3D bundle. The fibers provided the cell-laden hydrogel with typical topographic stimulus, and the collagen-coated surface exhibited biochemical cues. They synergically promoted myotube formation and myoblast differentiation [135]. Nanofiber yarn and hydrogel were assembled to prepare a composite scaffold with a core-shell structure (Figure 7(a)) [133]. The fiber yarn core comprised of PCL, polyaniline, and silk fibroin was fabricated through dry-wet electrospinning, which had the function of inducing 3D cellular alignment and elongation. The photocrosslinked PEG hydrogel was used as a shell to embed the cell-seeded yarns. This hydrogel shell showed the protection for cell proliferation and the 3D environment for cell arrangement. Moreover, the hydrogel with a similar stiffness of muscle tissue could prevent the random winding and twining of yarns during operation, thereby reducing the possibility of cell detachment. Finally, C2C12 myoblast cultured within this yarn/hydrogel core-shell scaffolds *in vitro* showed enhanced cellular alignment and myogenic differentiation, further proving the advantages and promising prospects of nanofiber/hydrogel composite scaffolds in the application of skeletal muscle regeneration (Figures 7(b) and 7(c)).

### 4.4. Nerve

Nerve injuries caused by diseases, trauma, or tumor operations can lead to loss of movement dysfunctions and extreme pains. Spontaneous regeneration produces unsatisfied recovery results, and the availability of donor's nerves limits the autologous nerve graft. Therefore, different kinds of neural tissue engineering scaffolds have become promising for nerve repair [136]. Among them, electrospun nanofibers with various mechanical and biochemical stimuli are often used. The physical properties of electrospun fibers, such as alignments [137], stiffness, and topography [138], and the biochemical properties, such as RGD and growth factor [139, 140], have been systematically studied. Also, conductive polymers (e.g., polyaniline [141]) and nanoparticles (e.g., graphene [142]) have been fabricated or added to the nanofibers to promote neural tissue regeneration [143, 144].

**Figure 8:**
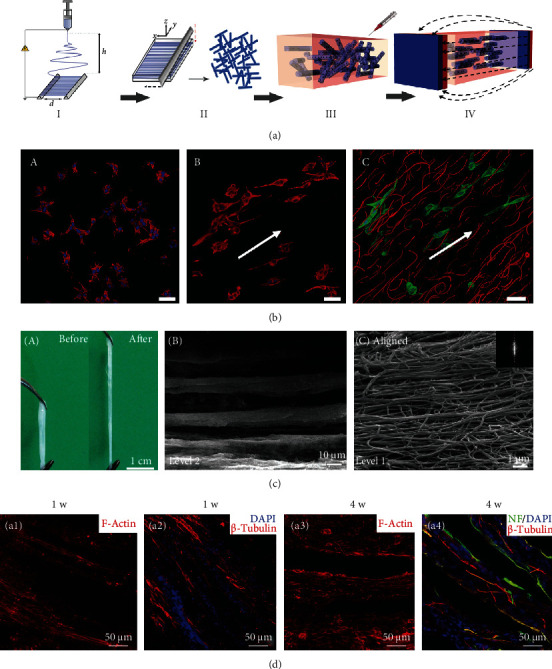
Electrospun nanofibers in 3D scaffolds and their effects on cell alignment and neural differentiation. (a) A schematic illustration of the fabrication process of Anisogel. Electrospinning of aligned fibers (step I). Short fibers forming by cryosectioning (step II). Randomly oriented short fibers mixed within the hydrogel precursor solution before gelation and applying the magnetic field (step III). Fiber orientation under low magnetic field and hydrogel crosslinking result in the Anisogel (step IV). (b) The ability of the Anisogel to direct cell growth. (A) Fibroblasts mixed within a fibrin gel without fibers, (B) fibroblasts mixed within a fibrin gel with short oriented fibers, and (C) fibroblasts elongate in the direction of the oriented fibers. Scale bars 50 *μ*m. Reproduced with permission from [80], Copyright © 2017, WILEY-VCH Verlag GmbH & Co. KGaA, Weinheim. (c, A) Electrospun-aligned fibrillar fibrin hydrogel with good flexibility. (B, C) Hierarchically aligned microstructure at different magnifications. (d) Immunofluorescence staining images of the longitudinal tissue section from the T8–T10 spinal cord segment at 1 and 4 weeks after scaffold implantation. Reused with permission from [149]. Copyright © 2016, Royal Society of Chemistry.

3D environment is critical for cell activities and cell-cell interactions, which affect cell differentiation and neo tissue regeneration [145–147]. It was reported that aligned nanofibers could provide physical cues for guiding neural differentiation in the 2D surface. Based on this principle, aligned nanofiber/hydrogel 3D composited scaffolds were designed, which exhibited practical nerve tissue engineering functions. An external magnetic field could control the alignment of short fibers suspended in the hydrogel before complete gelation of the matrix, which endowed the anisotropic property of this composite matrix. Cells encapsulated in the aligned fibers/hydrogel construct stretched F-actin filaments in the direction of fibers. The experiment confirmed that the neurons encapsulated in this matrix had a spontaneous electrical activity with calcium signals propagating along with the orientation of these iron oxide-loaded PLGA short fibers (Figures 8(a) and 8(b)) [80]. Similarly, an aligned fibrin nanofibrous hydrogel for peripheral nerve regeneration was prepared by electrospinning and self-assembly methods. This fibrous hydrogel/chitosan composite scaffold promoted the regrowth of axons *in vivo* [148]. In addition, due to the matrix stiffness which is another important biomechanical cue for cell differentiation, a fibrous and aligned fibrin hydrogel scaffold with soft elasticity was fabricated by electrospinning. This construct promoted the stem cells' neurogenic differentiation and rapid neurite outgrowth *in vivo* spinal cord injury model (Figures 8(c) and 8(d)) [149].

Furthermore, it has been reported that the hydrogel matrix can provide a protective barrier to shield the transplanted cell from a toxic environment, under which most of the transplanted cells will gradually die, resulting in minor effects on tissue regeneration [150]. Biomimetic multichannel silk conduits were prepared by an electrospinning technique followed by manual manipulation to create perineurium-like structures, mimicking the architecture of the native nerve [151]. Surrounded hydrogels prevented the cells from being directly exposed to the harsh environment and thus presented low oxygen and nutrient level, apoptotic cytokines, and some toxic reactive oxygen species. Therefore, electrospun nanofiber-based 3D composite scaffolds have exhibited improved controllability in mechanical properties, cell protection, and good performances *in vivo*, showing promising applications in neural tissue engineering.

### 4.5. Cardiac Tissue

As the major tissue for systole and diastole, the myocardium exhibits a hierarchical structure with aligned cells embedded into micropatterned and anisotropy ECM [27]. Therefore, electrospun nanofibers have been widely explored for myocardial tissue regeneration, which can mimic the myocardium anisotropic structure, direct the alignment of cardiomyocytes (CMs), and provide a suitable microenvironment for cell phenotype and neo tissue regeneration [152]. Electrospun nanofibers with different mechanical and biochemical properties for cardiac tissue engineering can be fabricated using different polymers, hybridization with other polymers, or nanoparticles, loading with growth factors or drugs.

**Figure 9:**
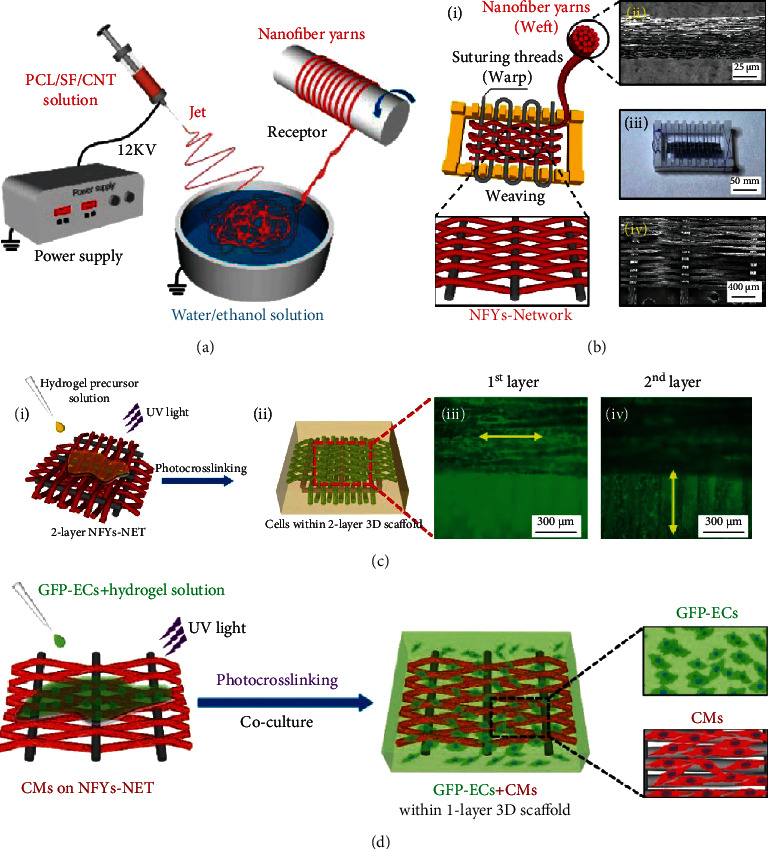
Nanofiber yarn/hydrogel composite 3D scaffolds with alignment, conductivity, and anisotropy for cardiac tissue engineering. Schematic illustration of (a) PCL/SF/CNT nanofiber yarns prepared by wet-dry electrospinning and (b) NFYs-NET scaffolds fabricated by a weaving technique. (c) 3D composite scaffold fabricated via encapsulating two layers of NFYs-NET with orthogonal orientation within UV crosslinked GelMA hydrogel (i). F-Actin (green) staining of CMs cultured on the NFYs-NET layer with horizontal direction (iii) and vertical direction (iv) within a 2-layer 3D scaffold. (d) Coculture of CMs and ECs within yarn/hydrogel 3D composite scaffolds. CMs were cultured on the NFYs-NET layer while ECs with green fluorescent protein were encapsulated within a hydrogel shell. Reproduced with permission from [67]. Copyright © 2017, American Chemical Society.

Strong mechanical properties of scaffolds are required to restore heart functions, such as systole. However, many studies have shown that CMs prefer the matrix with soft stiffness [153, 154]. Electrospun nanofiber-based 3D composite scaffolds afford an optimized approach for resolving this challenge. For example, the multiple aligned fiber layer-incorporated hydrogel scaffold was created through wet-dry electrospinning and photocrosslinking techniques, respectively (Figures 9(a) and 9(b)) [67]. The conductive nanofiber yarn network (NFY-NET) was fabricated with polycaprolactone, silk fibroin, and carbon nanotubes through wet-dry electrospinning. The hydrogel was made of biocompatible GelMA hydrogel, which had the function to provide a preferred 3D environment for cell differentiation, nutrition exchange, thickness increase, and mechanical protection (Figure 9(c)). CMs cultured on the NEYs-NET scaffolds showed more robust and synchronized beating than that cultured on the 2D glass surface. Furthermore, the scaffold's mechanical property and structure stability were improved after incorporating with GelMA hydrogels. CMs and endothelial cells (ECs) could be separately seeded on the surface of yarns and encapsulated in GelMA hydrogel for coculture, indicating the potential for using integrated cardiovascular organoids (Figure 9(d)).

Heart valve tissue engineering is a promising approach for injured or diseased heart value repairing. An electrospun fiber mesh with heterogeneous and anisotropic properties was created by textile techniques to mimic the native features of heart value tissue. After combining with cell-laden methacrylated hyaluronic acid/GelMA hydrogel, this composite scaffold exhibited better mechanical strength, enhanced proliferation, and balanced ECM remodeling against degradation and shrinkage compared with single materials [155]. The nanofiber composite scaffold can also be designed for coronary artery vessel regeneration, comprised of separate collagen and elastin fibers [156]. For example, an electrospun mesh was cut into a rectangular piece with the short edge parallel with the orientation of the fibers and rolled wrapping around a latex tube. Then, a hollow Teflon cylinder was placed around the wrapped tube as a mold, and a thrombin/fibrinogen-cell suspension was added into the structure. After crosslinking and removing the latex tube and Teflon cylinder, a fiber/hydrogel composite was left. This composite scaffold was biphasic in the mechanical property due to the different mechanical performance with the inner electrospun fibers and outer hydrogel, making it a potential construct for coronary artery vessel tissue engineering [157].

## 5. Conclusion and Future Perspectives

3D ENF-S represent a novel class of materials and have shown great promise for tissue engineering due to their intrinsic merits. These scaffolds with improved fibrous structure and thickness are superior to the 2D electrospun nanofiber membrane in cell infiltration and homogenous tissue regeneration. Besides, the tunable fibrous structure and mechanical properties of 3D ENF-S are attractive for tissue regeneration. Moreover, the ability to provide 3D biochemical and biophysical stimulus and cell protection from harsh environments is also present in electrospun nanofiber/hydrogel composite 3D scaffolds. Therefore, these 3D ENF-S have demonstrated effectiveness for cartilage and bone, tendon and ligament, skeletal muscle, nerve, and cardiac tissue engineering.

Despite the encouraging progress, utilizing 3D ENF-S for tissue engineering still faces many challenges. Ideally, bioinspired tissue engineering scaffolds should have highly ordered architecture because natural tissues or organs consist of multiscale hierarchical structures. Future studies focusing on the biomimetic hierarchical structure may bring additional functions for cell migration, proliferation, and neo tissue deposition. Moreover, it is necessary to study the interface between the fiber and porous scaffold/hydrogel matrix, which is related to the mechanical properties of the construct and affects cell activities and functions. It was reported that the interfacial bonding between fiber and hydrogel has the effect of promoting angiogenesis [158].

Finally, the long-term safety and performance of 3D ENF-S *in vivo* are the direction of future study in clinical transplantation. 3D ENF-S are usually prepared from two or more materials, and the synthetic polymers are generally electrospun in an organic solvent. Therefore, the toxicity of degradation products *in vivo* should be thoroughly evaluated. Also, the predesigned mechanical properties, hierarchical and anisotropic structures, and degradation profiles tested *in vitro* cannot reflect the results in the dynamic environment *in vivo*. All these performances will change with the scaffold degradation and the ingrowth of new tissue after implantation in the body. Therefore, the dynamic changes of the microenvironment *in vivo* should be taken into consideration when designing and preparing the 3D ENF-S with functional structure and properties.

## Data Availability

The figures and data supporting this review are from previously reported studies and datasets, which have been cited and obtained permissions.
